# Supplementary material for: From sequence to enzyme mechanism using multi-label machine learning
Source: BMC Bioinformatics. 2014 May 19;15:150. doi: 10.1186/1471-2105-15-150 (PMC4229970; doi:10.1186/1471-2105-15-150)
Supplement: Additional file 2 — Java code of ml2db. Additional file ml2db_code.tar.gz contains the Java source code to run the multi-label machine learning experiments and save the results to database. The code’s Javadoc is included. [file 1471-2105-15-150-S2.zip › additional file 2/ml2db/ml2db/doc/mulan/evaluation/measure/InformationRetrievalMeasures.html]

InformationRetrievalMeasures


JavaScript is disabled on your browser.


- Overview
- Package
- Class
- Use
- Tree
- Deprecated
- Index
- Help

- Prev Class
- Next Class

- Frames
- No Frames

- All Classes

- Summary:
- Nested |
- Field |
- Constr |
- Method

- Detail:
- Field |
- Constr |
- Method


mulan.evaluation.measure

## Class InformationRetrievalMeasures

- java.lang.Object
- - mulan.evaluation.measure.InformationRetrievalMeasures

- ---

    

  ```
  public class InformationRetrievalMeasures
  extends java.lang.Object
  ```

  Class for computing various information retrieval measures.

  Version:
  :   2012.05.29

  Author:
  :   Grigorios Tsoumakas

- - ### Constructor Summary

    Constructors

    Constructor and Description

    `InformationRetrievalMeasures()`
  - ### Method Summary

    Methods

    | Modifier and Type | Method and Description |
    | --- | --- |
    | `static double` | `fMeasure(double tp, double fp, double fn, double beta)` Computation of F-measure based on tp, fp, fn and beta. |
    | `static double` | `precision(double tp, double fp, double fn)` Computation of precision based on tp, fp and fn. |
    | `static double` | `recall(double tp, double fp, double fn)` Computation of recall based on tp, fp and fn. |
    | `static double` | `specificity(double tn, double fp, double fn)` Computation of specificity based on tn, fp and fn. |

    - ### Methods inherited from class java.lang.Object

      `equals, getClass, hashCode, notify, notifyAll, toString, wait, wait, wait`

- - ### Constructor Detail


    - #### InformationRetrievalMeasures

      ```
      public InformationRetrievalMeasures()
      ```
  - ### Method Detail


    - #### fMeasure

      ```
      public static double fMeasure(double tp,
                    double fp,
                    double fn,
                    double beta)
      ```

      Computation of F-measure based on tp, fp, fn and beta. We treat special
      cases with empty set predictions or/and ground truth as follows: (i) if
      the algorithm outputs the empty set and the ground truth is the empty
      set, then we consider F equal to 1 (ii) if the algorithm outputs the
      empty set and the ground truth is not empty, then we consider F equal to
      0 (iii) if the ground truth is empty and the algorithm does not output
      the empty set, then we consider F equal to 0 (iv) if neither the ground
      truth nor the algorithm's prediction is the empty set and their
      intersection is empty, then we consider F equal to 0.

      Parameters:
      :   `tp` - true positives
      :   `fp` - false positives
      :   `fn` - false negatives
      :   `beta` - controls the relative importance of recall versus precision

      Returns:
      :   the value of the f-measure


    - #### precision

      ```
      public static double precision(double tp,
                     double fp,
                     double fn)
      ```

      Computation of precision based on tp, fp and fn. We treat special cases
      with empty set predictions or/and ground truth as follows: (i) if the
      algorithm outputs the empty set and the ground truth is the empty set,
      then we consider precision equal to 1 (ii) if the algorithm outputs the
      empty set and the ground truth is not empty, then we consider precision
      equal to 0.

      Parameters:
      :   `tp` - true positives
      :   `fp` - false positives
      :   `fn` - false negatives

      Returns:
      :   the value of precision


    - #### recall

      ```
      public static double recall(double tp,
                  double fp,
                  double fn)
      ```

      Computation of recall based on tp, fp and fn. We treat special cases with
      empty set predictions or/and ground truth as follows: (i) if the
      algorithm outputs the empty set and the ground truth is the empty set,
      then we consider recall equal to 1 (ii) if the algorithm does not output
      the empty set and the ground truth is empty, then we consider recall
      equal to 0.

      Parameters:
      :   `tp` - true positives
      :   `fp` - false positives
      :   `fn` - false negatives

      Returns:
      :   the value of recall


    - #### specificity

      ```
      public static double specificity(double tn,
                       double fp,
                       double fn)
      ```

      Computation of specificity based on tn, fp and fn. We treat special cases
      with empty set predictions or/and ground truth as follows: (i) if the
      algorithm outputs the set of all labels and the ground truth is the set
      of all labels, then we consider specificity equal to 1 (ii) if the ground
      truth is the set of all labels and the algorithm does not output the set
      of all labels, then we consider specificity equal to 0.

      Parameters:
      :   `tn` - true negatives
      :   `fp` - false positives
      :   `fn` - false negatives

      Returns:
      :   the value of specificity


- Overview
- Package
- Class
- Use
- Tree
- Deprecated
- Index
- Help

- Prev Class
- Next Class

- Frames
- No Frames

- All Classes

- Summary:
- Nested |
- Field |
- Constr |
- Method

- Detail:
- Field |
- Constr |
- Method
